# Low occurrence of extended-spectrum cephalosporinase producing *Enterobacteriaceae* and no detection of methicillin-resistant coagulase-positive staphylococci in healthy dogs in Sweden

**DOI:** 10.1186/s13028-020-00516-4

**Published:** 2020-04-25

**Authors:** Stefan Börjesson, Lotta Gunnarsson, Annica Landén, Ulrika Grönlund

**Affiliations:** 1 Department of Biomedical and Clinical Sciences, Linköping University, 58183 Linköping, Sweden; 2 Department of Animal Health and Antimicrobial Strategies, National Veterinary Institute (SVA), 75189 Uppsala, Sweden; 3 AniCura, Vendevägen 89, 182 32 Danderyd, Sweden

**Keywords:** *bla*_CTX-M_, *bla*_CMY-2_, Canine, * E. coli*, Enterobacteriaceae, ESBL, MRSA, MRSP, pAmpC, *S. aureus*, S*. pseudintermedius*

## Abstract

Sweden has a long tradition of monitoring occurrence of antibiotic resistant bacteria in both animals and humans, but there currently is no organised and harmonized monitoring on carriage of Enterobacteriaceae producing extended-spectrum beta-lactamase (ESBL), plasmid-mediated AmpC beta-lactamase (pAmpC), or methicillin-resistant coagulase positive staphylococci e.g. methicillin-resistant *Staphylococcus aureus* (MRSA) and methicillin-resistant *Staphylococcus pseudintermedius* (MRSP) in dogs. The aim of the current study was therefore to determine the prevalence of ESBL/pAmpC producing Enterobacteriaceae and methicillin-resistant coagulase positive staphylococci in healthy dogs in Sweden, and to phenotypically and genotypically characterize any identified isolates. It was shown that 0.9% (95% confident interval 0.3–2.7%) of the dogs (n = 325) carried multi-resistant ESBL-producing *Escherichia coli*, but that no methicillin-resistant coagulase positive staphylococci could be detected. In conclusion, the occurrence of multi-drug resistant bacteria remains rare among healthy dogs in Sweden. In addition, the ESBL-producing *E. coli* identified showed genetic characteristics related to those reported from humans.

## Findings

Multi-resistant bacteria are an increasing threat to both animal and human health. Enterobacteriaceae producing extended-spectrum beta-lactamase (ESBL) or plasmid-mediated AmpC beta-lactamase (pAmpC), and methicillin-resistant coagulase positive staphylococci (MRCPS) are of significant concern. Regarding coagulase positive staphylococci, the main concern in human health is the emergence of methicillin-resistant *Staphylococcus aureus* (MRSA), while for companion animals, particularly among dogs, the concern is primarily methicillin-resistant *Staphylococcus pseudintermedius* (MRSP). Carriage of ESBL/pAmpC producing Enterobacteriaceae (ESBL/pAmpC-E) in dogs appears to vary between settings and countries. In Copenhagen, Denmark, 1.9% of dogs’ faecal deposits in public gardens were positive for ESBL-producing *Escherichia coli* (ESBL-EC) and in Paris, France, 18.5% of dogs carried ESBL-EC, while 81.8% of dogs carried ESBL-EC in Faisalabad, Pakistan [1–3]. Other nationwide studies reported ESBL/pAmpC-E carriage rates of 9.0% in United Kingdom and 22.2% in Turkey, while in the Netherlands, 10.6% of dogs were reported to carry ESBL-E [4–6]. Occurrence of MRCPS in dogs generally appears to be lower with reports around 0–3% positive dogs [7–10].

There are several reasons why it is important to have an up-to-date data on national occurrence of ESBL/pAmpC-E and MRCPS in healthy dogs: (i) these dogs are potential high-risk patients in veterinary settings due to the risk of transmission and if becoming diseased, for the risk of failure when treated with antibiotics (ii) sentinel data are essential information when investigating outbreaks in hospitals and clinics to determine if the infectious agent causing the outbreak is acquired in the community or in the hospital and (iii) the zoonotic aspect of carriage with positive dogs functioning as reservoirs or vectors for community-acquired ESBL/pAmpC-E and MRCPS in humans.

In Sweden, there is a relative low usage of antibiotics to dogs and Sweden is generally considered to have a favourable status regarding occurrence of antibiotic resistant bacteria [11]. However, there is no current surveillance on carriage of ESBL/pAmpC-E and MRCPS in dogs or any other companion animals. In addition, the latest screening for ESBL/pAmpC-E and MRCPS in dogs was conducted in 2012 and included only 84 dogs [11]. In that study, only one dog was found to carry pAmpC-producing *E. coli* (pAmpC-EC) and no MRCPS were detected. The objective of the current study was to provide up-to-date data on prevalence and types of ESBL/pAmpC-E and MRCPS among healthy dogs in Sweden.

The present study was conducted from May 2017 to May 2018 as a collaboration between The National Veterinary Institute (SVA), Sweden, and AniCura, Sweden. Samples were collected from 325 healthy dogs > 1 year of age. The samples were taken at a visit at one of eight AniCura animal hospitals in Sweden that participated in the study. Dogs included in the study visited the hospitals for vaccinations or other standard procedures for healthy dogs like x-rays of hips or elbows, blood donations, etc. Two samples were collected per dog using Copan ESwab™; a rectal swab for ESBL/pAmpC-E isolation and a pooled swab from labial comissure, pharynx, perineum and any wounds (if present) for MRCPS screening. The swabs were sent to one of five participating AniCura laboratories. For ESBL/pAmpC-E, 0.5 mL of suspension fluid was added to 4.5 mL buffered-peptone-water and incubated at 36 ± 1 °C overnight. After incubation, 10 µL of the enrichment broth was plated on MacConkey Agar with 1 μg/mL cefotaxime and incubated at 37 °C, 18–22 h. For MRCPS, 0.2 mL of suspension fluid was added to 4.8 mL trypticase soy broth with 4% NaCl, 1% mannitol and 10 mg/L aztreonam and incubated at 36 ± 1 °C overnight. After incubation, 10 µL of the enrichment broth was plated on Mannitol salt agar with a 1 μg oxacillin MASTDISCS^®^ disc (MAST group, Bootle, England) and Brilliance MRSA-2-agar (Oxoid AB, Malmö, Sweden) and incubated at 36 ± 1 °C, 18–24 h. One random colony was selected from positive agar plates and sent to SVA for confirmation. At SVA, species identification was performed using Bruker MALDI Biotyper System and suspected ESBL/pAmpC-E isolates were thereafter confirmed phenotypically and tested for antibiotic susceptibility using Sensititre™ EUVSEC and EUVSEC2 microdilution panels (Thermo Fischer Scientific, Waltham, MA USA) while MRCPS was confirmed by polymerase chain reaction [12]. Verified isolates, including the isolate from the 2012 screening, were subjected to next-generation-sequencing using Illumina based technologies and subjected to alignment and bioinformatic analyses as previously described [13]. Serotypes were determined using SerotypeFinder (https://cge.cbs.dtu.dk/).

Three out of 325 dogs were found to carry ESBL-EC (0.9%; 95% confident interval 0.3–2.7% using Wilson Score interval) (Table 1). The prevalence of ESBL-E in Swedish dogs was lower than that reported from other European countries [2–5], but more in-depth comparisons are difficult to make because of general application of different methodologies. However, a Dutch study conducted in 2014–2016 used similar methods as the current study and they found that 10.6% of the 555 dogs carried ESBL-E [4]. In addition, they also reported that 3.8% of the owners were carriers, which was slightly lower than the 4.5% prevalence rate reported from the Dutch general population [4, 14]. The carriage rate in Dutch citizens is comparable to 4.7% reported from Sweden in 2013 [15], so it is interesting to note the large difference between carriage rates in Dutch and Swedish dogs. One potential explanation for the difference might be that use of extended spectrum cephalosporins (ESCs) is very limited in Sweden, while in the Netherlands, ESCs, i.e. cefovecin, was frequently used for companion animals [10, 16]. The low usage in Sweden is partly due to a regulation implemented in 2013 which limits the veterinarians’ right to prescribe ESCs, stating that these can only be used if alternative choices cannot be expected to be successful (The Swedish Board of Agricultures regulations on drugs and drug usages (In Swedish), SJVFS 2013:42, https://www.jordbruksverket.se). In the current study, all three isolates were also multi-resistant, i.e. resistant to > 2 antibiotic classes, and carried multiple genes encoding antibiotic resistance (Table 1). Had these strains been the causative agent of an infection, there would have been no or only a limited number of treatment options available for the handling veterinarian due to legalisation and antibiotic treatment policies in Sweden.

**Table 1:** Genotypic and phenotypic characteristics in three *Escherichia coli* isolated from Swedish dogs from May 2017 to May 2018, and from a screening study conducted in 2012

Year	Isolate	Genes encoding ESBL	MLST	Serotype	Plasmid replicon types^a^	Genes encoding antibiotic resistance	Antibiotic resistance	MIC for Az^b^
2012	CH79ctx	*bla*_CMY-2_	ST38	O7:H18	colMG828, B/O/K/Z, FIB FII, p0111	*mdf*A	Am, Cx, Cm	4
2017	ACS2	*bla*_CTX-M-1_	ST4496	O8:H28	FIA, HI1A, HI1B, Q1	*aac*(3)-IIb, *aad*A2, *aph*(3″)-Ib, *aph*(6)-Ib, *cat*A1, *dfr*A12, *mdf*A, *sul*1, *sul*2, *tet*A, *mph*A	Am, Cx, Cm, Chl, Gm, Su, Tm, Tc	8
	ACS5	*bla*_CTX-M-55_	ST354	O1:H34	col156, FIA, FIB, FII, Q1	*bla*_TEM-1B_, *aac*(3)-IId, *aad*A5, *ant*(3″)-Ia, *aph*(3″)-Ib, *aph*(6)-Ib, *cat*A1, *dfr*A17, *mdf*A, *mph*A, *sul*1, *sul*2, *tet*A, *tet*D	Am, Cx, Cm, Chl, Ci, Gm, Nal, Su, Tm, Tc	16
	ACS6	*bla*_CTX-M-27_	ST131	O25:H4	col156, col8282, FIA, FIB, FII	*aad*A5, *ant*(3″)-Ia, *aph*(3″)-Ib, *aph*(6)-Id, *dfr*A17, *mdf*A, *mph*A, *sul*1, *sul*2, *tet*A	Am, Cx, Cm, Ci, Nal, Su, Tm, Tc	32

All isolates were tested for susceptibility against ampicillin (Am), azithromycin (Az), cefotaxime (Cx), ceftazidime (Cm), chloramphenicol (Chl), ciprofloxacin (Ci), colistin (Co), gentamicin (Gm), meropenem (Me), nalidixic acid (Nal), kanamycin (Km), sulfamethoxazole (Su), trimethoprim (Tm), tigecycline (Tg) and tetracycline (Tc). Isolates were defined as resistant if minimum inhibitory concentration (MIC) was above the epidemiological cut-off values (ECOFFs) defined by EUCAST

^a^Plasmidfinder (https://cge.cbs.dtu.dk/) cannot differentiate between incB, incO, incK, incZ

^b^No ECOFF defined by EUCAST

In previous European studies, being fed raw meat and/or raw pet food was a risk factor for ESBL/pAmpC-E carriage in dogs [4, 5]. In addition, the *bla*_CTX-M-1_, carried on incI1 or incK plasmids are frequently identified in the European dog population, which is also common in the European poultry production [2–4, 17]. In the current study, the *bla*_CTX-M-1_ was identified but the incI1 or incK plasmid could not be detected in this isolate (Table 1). The *bla*_CTX-M-1_ isolate was also multi-resistant, which is inconsistent with the *bla*_CTX-M-1_ isolates identified in Swedish broilers and the multi-locus sequence type (MLST) ST4496 has not been identified in Swedish poultry [18, 19]. However, the 2012 screening isolate carried a *bla*_CMY-2_ gene, belonged to ST38 and was positive for incB/O/K/ZB plasmid replicon (Table 1). ST38 *E. coli* carrying incK + *bla*_CMY-2_ are common on Swedish poultry meat [18], and raw feed containing poultry meat in Sweden has been shown to be contaminated with *E. coli* carrying *bla*_CMY-2_ [20]. In addition to feed, another source for ESBL/pAmpC-E occurrence in dogs could be transmissions from humans. Earlier studies have described that, in addition to *bla*_CTX-M-1_, *E. coli* with *bla*_CTX-M-15_ and *bla*_CTX-M-14_ are common from dogs and these genes are also the most frequent genes detected in humans [1–6, 14, 15, 21]. It has also been shown that ESBL/pAmpC-E can be shared between owners and dogs [4, 22]. For example, a Swedish study showed that in 22 households with dog-owners previously identified with ESBL/pAmpC-EC, identical *bla*_CMY-2_ and *bla*_CTX-M-27_ positive *E. coli* isolates were confirmed in two households in both humans and dogs [22]. In the current study, the *E. coli* isolates carried *bla*_CTX-M-55_ and *bla*_CTX-M-27_ in addition to the *bla*_CTX-M-1_ (Table 1). Among humans in Sweden, the genes *bla*_CTX-M-15_, *bla*_CTX-M-14_, *bla*_CTX-M-27_ and *bla*_CTX-M-1_ are the most frequently detected, but the *bla*_CTX-M-55_ is rare [15]. Furthermore, the *bla*_CTX-M-55_ has been identified from wild-birds and pigs in Sweden, confirming that it occurs in Sweden and within different settings [10, 23, 24]. Additionally, the *bla*_CTX-M-27_ isolate probably is linked to humans because it was identified as an O25:H4-ST131, which is a pandemic clone, both as non-ESBL and as an ESBL-producer in humans [21]. The *bla*_CTX-M-27_ O25:H4-ST131 isolate also was positive for incF-plasmids (Table 1), including incFII which has been strongly linked to the carriage of *bla*_CTX-M-27_ in human O25:H4-ST131 [15, 21].

None of the 325 dogs in the current study carried MRCPS, but the lack of MRCPS in dogs in Sweden was not unexpected since previous studies have described no or low occurrence in healthy dogs [7–9, 11]. Additionally, both MRSA and MRSP are rare among clinical cases in dogs in Sweden [11]. In fact, only a handful of MRSA cases are reported each year while findings of MRSP are more common with 40–60 cases usually reported annually. In contrast to this study, a recent study from neighbouring Finland on guide dogs described an MRSP occurrence of 3% [10].

The current study demonstrates that occurrence of multi-drug resistant bacteria remains rare among dogs in Sweden, with only 0.9% of dogs carrying ESBL-producing *E. coli* and no dogs were identified with methicillin-resistant coagulase-positive staphylococci. Based on molecular typing, the results indicated that the occurrence of ESBL-producing *E. coli* in dogs in Sweden could be due to transmission of strains from humans.

## Data Availability

The datasets generated and analysed during the current study are available from the corresponding author on reasonable request. Sequence reads for the four isolates have been deposited in the European Nucleotide Archive under the accession number PRJEB35649.

## Abbreviations

ECOFF: Epidemiological cut-off value
ESBL: Extended-spectrum beta-lactamase
ESBL/pAmpC-E: ESBL/pAmpC producing Enterobacteriaceae
ESBL-EC: ESBL-producing *E. coli*
ESC: Extended spectrum cephalosporins
MIC: Minimum inhibitory concentration
MLST: Multilocus sequence typing
MRCPS: Methicillin-resistant coagulase positive Staphylococci
MRSA: Methicillin-resistant *S. aureus*
MRSP: Methicillin-resistant *S. pseudintermedius*
pAmpC: Plasmid-mediated AmpC beta-lactamase
pAmpC-EC: pAmpC-producing *E. coli*
